# Association between serum cotinine and α-Klotho levels among adults: Findings from the National Health and Nutrition Examination Survey 2007–2016

**DOI:** 10.18332/tid/144622

**Published:** 2022-06-14

**Authors:** Yu Yao, Ying Long, Fa-Wang Du, Yong Zhao, Xiao-Bin Luo

**Affiliations:** 1 Department of Respiratory and Critical Care Medicine, Suining Central Hospital, Suining, China

**Keywords:** smoking, serum cotinine, α-Klotho

## Abstract

**INTRODUCTION:**

Serum cotinine is a sensitive and specific marker of tobacco smoke exposure. α-Klotho is an anti-ageing molecule, which plays an important role in several diseases. We aimed to examine the association between smoke exposure indicated by the serum cotinine and α-Klotho levels, as previous reports regarding the level of α-Klotho in smokers have been inconsistent.

**METHODS:**

This secondary dataset analysis included 9833 participants (aged 40–79 years; 47.0% females and 53.0% males) from the US National Health and Nutrition Examination Survey 2007–2016. Independent variables were serum cotinine level, age, sex, race, body mass index (BMI), and alcohol consumption. The outcome variable was serum α-Klotho level. Multiple linear regression analysis was used to examine the association between serum cotinine and α-Klotho levels.

**RESULTS:**

The serum cotinine level was negatively associated with the α-Klotho level (*β*= -0.107, 95% CI: -0.155 to -0.059, p<0.0001) after adjusting for age, BMI, sex, race, and alcohol consumption. The α-Klotho level in participants with cotinine ≥3 ng/mL decreased by 44.514 pg/mL (p<0.0001) compared to that in participants with cotinine <3 ng/mL. There is a non-linear relationship between serum cotinine and α-Klotho levels. The piecewise linear models indicated a significant threshold effect between serum cotinine and α-Klotho levels. On the left of the inflection point (cotinine <130 ng/mL), the serum cotinine level increased with decreased α-Klotho level (*β*= -0.519, 95% CI: -0.682 to -0.356). On the right of the inflection point (cotinine ≥130 ng/mL), the serum cotinine level increased with increased α-Klotho level (*β*=0.085, 95% CI: 0.000 to 0.170).

**CONCLUSIONS:**

Based on our study results, serum cotinine level was associated with the serum α-Klotho level.

## INTRODUCTION

More than 1.1 billion people are smokers, and 0.5 billion people have died from smoking worldwide in 2012^1^. Smoking is one of the most common risk factors for many diseases, including cancer, chronic obstructive disease, and cardio-cerebrovascular diseases, which are also considered ageing-related diseases^2,3^. In addition, the lifespan of smokers is 10 years less than that of never smokers^2^.

α-Klotho is an anti-ageing molecule, which is expressed in various tissues, including the kidney, lung, choroid plexus, and skeletal muscle^4,5^. The decrease in Klotho levels contributes to the occurrence and development of many diseases^6-8^. Serum cotinine is a sensitive and specific marker of smoking, which has proven useful in estimating the risks associated with smoking status^9^.

The relationship between smoking and α-Klotho levels has been reported in many studies, and cigarette smoke induces airway inflammation by downregulating Klotho levels in the airway epithelium in chronic obstructive pulmonary disease (COPD)^10^. Smoking cessation significantly decreases serum α-Klotho levels and might be a compensatory response to smoking stress. Previous studies have shown that, in healthy men, the serum α-Klotho level was significantly higher in smokers than in never smokers^11^, and that α-Klotho level was lower in smokers without infection during preterm gestations^12^. However, these results are not consistent; therefore, the purpose of this study was to explore the association between serum cotinine and α-Klotho levels in participants from the National Health and Nutrition Examination Survey (NHANES) 2007–2016. To the best of our knowledge, this is the first study to examine the association between cotinine and α-Klotho levels.

## METHODS

### Study design

This was a cross-sectional secondary dataset analysis of data from the US NHANES, and it was conducted by both the Center for Disease Control and the National Center for Health Statistics (NCHS). Interviews and physical examinations were completed on a nationally representative sample of non-institutionalized individuals in the United States. A multistage stratified sampling design with oversampling for certain subgroups was used in the survey. Participants completed household surveys, including questionnaires about demographic characteristics and health history and provided blood and urine samples during physical examinations. Data were collected for a two-year survey cycle. All the participants provided written informed consent, and the study was approved by the NCHS Ethics Review Board.

### Participants

Our sample included participants aged 40–79 years from the NHANES 2007–2016, because data on α-Klotho were collected only during this period and the level of α-Klotho in middle-aged and elderly people is more susceptible. To reduce the bias in exploring the independent associations between serum cotinine and α-Klotho levels, we excluded individuals who had coronary heart disease, malignancy, stroke, liver condition, congestive heart failure, heart attack or weak/failing kidneys, as these disease may affect α-Klotho levels^13-17^. In total, 9833 participants with cotinine and α-Klotho data were enrolled for the final analysis. A serum cotinine cut-off value of 3 ng/mL was used to define the smoking status^9^. Active smokers were defined as participants who smoked >100 cigarettes during their lifetime and who currently smoked. We defined passive smokers as participants who did not actively smoke but were exposed to tobacco smoke either at home or in the workplace during the 7 days. Non-smokers were defined as participants who reported smoking <100 cigarettes during their lifetime, did not smoke currently, and were not exposed to environmental tobacco smoke.

### Variables

α-Klotho level was measured using a commercially available enzyme-linked immunosorbent assay (ELISA) kit produced by IBL International, Japan. The reference range was 285.8–1638.6 pg/mL, with a mean of 698.0 pg/mL^18^. Serum cotinine levels were measured using an isotope-dilution high-performance liquid chromatography/atmospheric pressure chemical ionization tandem mass spectrometric method. α-Klotho level was specified as the dependent variable.

Regarding the covariates, continuous variables included age and body mass index (BMI); categorical variables included sex, race (Mexican American/Other Hispanic /Non-Hispanic White/Non-Hispanic Black/other race), and alcohol consumption (minimum of 12 alcoholic drinks/year). Obesity was defined by BMI (≥30 kg/m^2^). Data regarding α-Klotho, serum cotinine, age, BMI, sex, alcohol consumption, and race are available in the NHANES dataset (https://www.cdc.gov/nchs/nhanes/index.htm).

### Statistical analysis

All statistical analyses were performed using the R-project (http://www.R-project.org) and EmpowerStats (http://www.empowerstats.com). Continuous variables were compared by T-test. Full sample 2-year MEC exam weight (WTMEC2YR) was divided by the number of cycles to recalibrate; then, all estimates were calculated using these recalibrated weights following the analytical guideline edited by the NCHS, and two multiple linear regression models were constructed with adjustment for possible baseline data imbalances: crude model – no covariates were adjusted; and adjusted model – age, sex, BMI, alcohol consumption, and race were adjusted. To evaluate the consistency of subgroups, subgroup and interaction analyses were performed to examine the association between serum cotinine tertiles (cut-off value was 3 ng/mL) and α-Klotho stratified by race (Mexican American vs other Hispanic vs non-Hispanic White vs non-Hispanic Black vs other race), sex (male vs female), age (<60 years vs ≥60 years), obesity (obese vs non-obese), and alcohol consumption (≥12 alcoholic drinks/year vs <12 alcoholic drinks/year). We performed a test for linear trends by entering the cut-off value for cotinine level (3 ng/mL). A smooth curve fitting was conducted to examine whether an independent variable was partitioned into intervals, and segmented regression and log-likelihood ratio tests were performed to determine whether a threshold existed.

## RESULTS

In 9833 participants who were enrolled (aged 40–79 years; 53.0% males, 39.9% non-Hispanic White), 76.1% of participants had serum cotinine level <3 ng/mL. Compared to participants with cotinine level <3 ng/mL, those with cotinine level ≥3 ng/mL had significantly lower BMI, proportion of females, proportions of non-smokers and passive smokers, age, and α-Klotho levels, with significantly higher levels of alcohol consumption, proportion of active smokers and proportions of non-Hispanic White and Black individuals (Table 1).

**Table 1:** Baseline characteristics of participants, NHANES 2007–2016, USA, (N=9833)

*Characteristics*	*Cotinine levels (ng/mL)*			*p^c^*
	*Total*	*<3 ng/mL*	*≥3 ng/mL*	
	*9833*	*n=7483 (76.1%)*	*n=2450 (23.9%)*
**BMI** (kg/m^2^)		29.50 ± 0.13	28.38 ± 0.19	<0.0001
**Sex** (%)^a^				
Male	4619	43.61	57.44	<0.0001
Female	5214	56.39	42.56	
**Age** (years)^a^		54.90 ± 9.96	52.27 ± 8.84	<0.0001
**At least 12 alcoholic drinks/year** (%)				
Yes	6420	69.85	81.94	
No	2653	23.59	10.86	<0.0001
Missing	760	6.56	7.20	
**Race** (%)^a^				
Mexican American	1699	8.01	5.41	
Other Hispanic	1154	5.27	3.79	
Non-Hispanic White	3924	70.97	73.39	<0.0001
Non-Hispanic Black	2014	8.64	13.12	
Other race	1042	7.11	5.29	
**α-Klotho** ^b^		863.99 ± 5.87	822.87 ± 8.163	<0.0001
**Active smokers**, n (%)	1863	64 (0.86)	1799 (76.55)	
**Passive smokers**, n (%)	5164	4824 (64.47)	340 (14.47)	<0.0001
**Non-smokers**, n (%)	2806	2595 (34.68)	211 (8.98)	

a Estimated using full sample 2-year MEC exam weight from NHANES.

b Values reported as the mean ± SD for continuous variables.

c p-value calculated by ANOVA for continuous variables and chi-squared test for categorical variables. BMI: body mass index.

In the crude model, there was a significant negative and unadjusted association between serum cotinine and α-Klotho levels (*β*= -0.099, 95% CI: -0.145 to -0.052, p<0.0001). The trend remained significant among the different cotinine level categories (p<0.001). In the adjusted model, the serum cotinine level was negatively associated with the α-Klotho level (*β*= -0.107, 95% CI: -0.155 to -0.059, p<0.0001); for every one-unit increase in the serum cotinine level, the α-Klotho level decreased by 0.107 pg/mL. Compared to the α-Klotho level in participants with a cotinine level <3 ng/mL, that in participants with cotinine level of ≥3 ng/mL decreased by 44.514 pg/mL (Table 2). The association between the α-Klotho and serum cotinine levels was consistent across subgroups stratified by race, sex, obesity, and alcohol consumption. However, in the subgroup analyses stratified by race, a negative association was not present in the Mexican American group, active smokers’ group and non-smokers’ group. The effect modification was evident with regard to participants aged <60 years who had a significant decrease in the α-Klotho level and an increase in the serum cotinine level compared with participants aged ≥60 years (interaction p=0.14) (Table 3).

**Table 2:** Association of serum cotinine and α-Klotho assessed by multiple linear regression models, NHANES 2007–2016 , USA (N=9833)

*Serum cotinine*	*Total (n=9833)*	*Crude model β (95% CI)*	*p*	*Adjusted model^a^ β (95% CI)*	*p*
Cotinine <3 ng/mL	7483	Reference		Reference	
Cotinine ≥3 ng/mL	2450	-41.124 (-59.022 to -23.226)	<0.0001	-44.514 (-62.538 to -26.489)	<0.0001
Cotinine levels (ng/mL)		-0.099 (-0.145 to -0.052)	<0.0001	-0.107 (-0.155 to -0.059)	<0.0001
p for trend		<0.001		<0.001	

a Adjusted for age, sex, BMI, race, and alcohol consumption. CI: confidence interval.

**Table 3:** Association between serum cotinine tertiles and α-Klotho by subgroup and interaction analyses, NHANES 2007–2016, USA (N=9833)

*Subgroups*	*Total (n=9833)*	*β (95%CI)*	*p*	*Interaction p*
Mexican American	1699	1.061 (-34.996 to 37.118)	0.85	
Other Hispanic	1154	-46.250 (-90.370 to -2.130)	0.04	
Non-Hispanic White	3924	-36.340 (-57.198 to -15.482)	0.0006	
Non-Hispanic Black	2014	-95.690 (-132.839 to -58.542)	<0.0001	
Other race	1042	-86.751 (-136.070 to -37.432)	0.0006	0.0002
Male	4619	-29.532 (-46.856 to -12.208)	0.0008	0.60
Female	5214	-60.027 (-82.157 to -37.897)	<0.0001	
<60 years	5814	-54.213 (-71.486 to -36.940)	<0.0001	0.14
≥60 years	3719	-7.051 (-31.640 to -17.537)	0.57	
Obese	3905	-60.100 (-78.319 to -41.882)	<0.0001	0.24
Non-obese	5928	-26.812 (-48.808 to -4.816)	0.02	
≥12 alcoholic drinks/year	6420	-42.321 (-58.518 to -26.124)	<0.0001	0.40
<12 alcoholic drinks/year	2653	-38.084 (-74.186 to -1.983)	0.04	
Active smokers	1863	36.541 (-3.230 to 76.312)	0.07	
Passive smokers	5164	-63.429 (-94.971 to -31.888)	<0.0001	
Non-smokers	2806	-55.235 (-142.869 to 32.399)	0.22	

**Figure 1:**
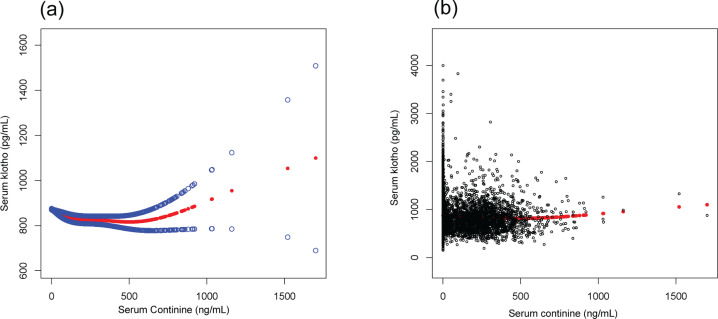
(a) The smooth curve of the relationship between serum Klotho and serum cotinine after adjusting for age, sex, body mass index (BMI), race, and alcohol consumption. (b) The scatter curve of the relationship between serum Klotho and serum cotinine after adjusting for the aforementioned variables. The blue lines are 95% CI and red line is a smooth curve fitting line

We found that the relationship between serum cotinine and α-Klotho levels was non-linear after adjusting for age, BMI, sex, race, and alcohol consumption (Figure 1). We found that the inflection point was 130 ng/mL, using a multiple two-piecewise linear regression model. On the left of the inflection point (cotinine <130 ng/mL): *β*= -0.519, 95% CI: -0.682 to -0.356, and p<0.0001. The level of α-Klotho was increased with the decrease of serum cotinine. On the right of the inflection point (cotinine ≥130 ng/mL): *β*=0.085, 95% CI: 0.000 to 0.170, and p=0.049. The level of α-Klotho was increased with the increase of serum cotinine (Table 4).

**Table 4:** Association between serum cotinine and α-Klotho levels by segmented regression and log-likelihood ratio test, NHANES 2007–2016, USA (N=9833)

*Serum cotinine*	*Total (n=9833)*	*Crude model*	*p*	*Adjusted model^a^*	*p*
Cotinine <130 ng/mL	8180	-0.487 (-0.650 to -0.323)	<0.0001	-0.519 (-0.682 to -0.356)	<0.0001
Cotinine ≥130 ng/mL	1653	0.084 (0.002 to 0.169)	0.055	0.085 (0.000 to 0.170)	0.049

a Adjusted for age, sex, BMI, race, and alcohol consumption. BMI: body mass index.

## DISCUSSION

As previous reports regarding α-Klotho levels in smokers have been inconsistent, we aimed to examine the association between smoke exposure indicated by serum cotinine and α-Klotho levels. In this analysis of a large, diverse, and nationally representative sample, we found that the serum cotinine level was associated with the α-Klotho level. However, this association was not significant in Mexican Americans, and after adjusting for confounding variables, there was a threshold effect and non-linear relationship between serum cotinine and α-Klotho levels. The threshold of serum cotinine was 130 ng/mL. These findings provide evidence for the association between ageing and smoking.

Cotinine is a metabolite from nicotine in the liver, and because of its long half-life, it is used as a biomarker for tobacco smoke exposure^9^. The relationship between smoking and α-Klotho levels has been proven by several studies; however, these results remain mixed. Among patients without infection, the α-Klotho level was decreased in female smokers with singleton gestations between 20 and 34 weeks^12^. Likewise, the Klotho level in the quadriceps was lower in current smokers^19^. In contrast, a study that enrolled 80 participants (40 smokers vs 40 never smokers) suggests that serum α-Klotho levels in never smokers were low, whereas they increased significantly in smokers^11^. In addition, cessation of cigarette smoking can decrease α-Klotho levels^20^. In our study, we found that the α-Klotho level increased with an increase in the cotinine level when the cotinine level was ≥130 ng/mL and decreased with the increase in the cotinine level when cotinine was <130 ng/mL. There may be a threshold effect due to a compensatory response to smoke exposure between smoking and α-Klotho levels. In our study, we observed no significant association between smoking and α-Klotho levels in Mexican Americans and the effect of serum cotinine tertiles on α-Klotho levels was affected by race. Previous studies demonstrated that serum cotinine levels in Whites are lower than those of Blacks at similar levels of tobacco exposure. Racial differences are related to different tobacco brands, differences in pharmacokinetics related to cytochrome P450 activity (CYP2A6), and smoking methods/habits^21^. In our study, we only observed significant association between smoking and α-Klotho levels in participants aged <60 years; α-Klotho is an anti-ageing protein, and the level of serum Klotho declines with ageing^22^. These findings illustrate that racial differences play an important role in smoking and ageing. Besides, we only found that there was a significant association between serum cotinine tertiles and α-Klotho levels in passive smokers. There may be discrepancy between self-reported smoking status and real smoking status.

Smoking is considered an important accelerator of the ageing process, which involves complex mechanisms and various pathogeneses^6^. Several studies have suggested that there is an association between COPD and rapidly increasing ageing^23^. Many studies have confirmed the association between COPD and Klotho levels; increasing soluble extracellular Klotho levels play a protective role in pulmonary epithelial cell death induced by cigarette smoke to reduce the risk of developing COPD^24^. Li et al.^25^ reported that inflammation induced by cigarette smoke extract decreased Klotho levels in alveolar macrophages, and that Klotho plays a role in maintained inflammation of the lungs, and that Klotho can inhibit cigarette smoke-induced autophagy, as one of the pathogeneses of COPD^26^. Klotho was downregulated by cigarette smoke in the airway epithelium from a murine COPD model and cultured human bronchial epithelial cells^10,27^. These are potential mechanisms by which smoking has an obvious effect on serum α-Klotho levels.

### Strengths and limitations

There are several limitations to this study when considering its implications and results. As a cross-sectional study, its temporality and residual confounding were some of the limitations. Another limitation of the study is that the single measurement of serum cotinine may not be able to fully capture the chronic secondhand and thirdhand smoke exposure. Despite these limitations, this study provides insight into the association between serum cotinine and α-Klotho levels in adults aged 40–79 years.

This study has many strengths, including large sample size, use of a representative, multiracial population, and better generalizability to the US population.

## CONCLUSIONS

Our study suggests that there is a non-linear relationship between serum cotinine and serum α-Klotho levels. As the extent of smoke exposure increased, the level of α-Klotho decreased first and then increased. Further studies are required to determine the underlying mechanisms of this compensatory response between serum cotinine and serum α-Klotho. These results suggested that smoking may induce aging by regulating α-Klotho levels.

## Data Availability

The data supporting this research are available from the authors on reasonable request.
